# Roles of Ferroptosis in Cardiovascular Diseases

**DOI:** 10.3389/fcvm.2022.911564

**Published:** 2022-05-23

**Authors:** Yuting Guo, Wei Zhang, Xinger Zhou, Shihao Zhao, Jian Wang, Yi Guo, Yichao Liao, Haihui Lu, Jie Liu, Yanbin Cai, Jiao Wu, Mingzhi Shen

**Affiliations:** ^1^The Second School of Clinical Medicine, Southern Medical University, Guangzhou, China; ^2^Department of Cardiology, Hainan Hospital of Chinese PLA General Hosptial, Hainan Geriatric Disease Clinical Medical Research Center, Hainan Branch of China Geriatric Disease Clinical Research Center, Hainan, China; ^3^Department of Cardiology, Second Medical Center, PLA General Hospital, Beijing, China; ^4^Department of Cardiology and Laboratory of Heart Center, Zhujiang Hospital, Southern Medical University, Guangzhou, China; ^5^Department of Cell Biology, National Translational Science Center for Molecular Medicine, Fourth Military Medical University, Xi'an, China

**Keywords:** ferroptosis, atherosclerosis, acute myocardial infarction, cardiomyopathy, heart failure

## Abstract

Ferroptosis is an iron-dependent regulated cell death characterized by lipid peroxidation and iron overload, which is different from other types of programmed cell death, including apoptosis, necroptosis, autophagy, and pyroptosis. Over the past years, emerging studies have shown a close relation between ferroptosis and various cardiovascular diseases such as atherosclerosis, acute myocardial infarction, ischemia/reperfusion injury, cardiomyopathy, and heart failure. Herein, we will review the contributions of ferroptosis to multiple cardiovascular diseases and the related targets. Further, we discuss the potential ferroptosis-targeting strategies for treating different cardiovascular diseases.

## Introduction

Cardiovascular diseases include hypertension, atherosclerosis, acute myocardial infarction (AMI), arrhythmia, cardiomyopathy, valvular heart diseases, congenital cardiovascular diseases and heart failure (1), which are the leading causes of disability and death in the world (2). Cardiomyocyte death is a basic pathological process in the progression of cardiovascular diseases. Understanding the mechanism of cardiomyocyte death can provide support for protecting cardiac function.

Ferroptosis, which was proposed by Dixon et al. (3), is a non-apoptotic form of cell death. Ferroptosis is characterized by lipid peroxidation and iron overload. Its morphological features mainly involve mitochondrial changes encompassing mitochondria shrinkage, increased mitochondria membrane density, crista destruction, and outer membrane rupture, but not nucleus morphological changes. Ferroptosis is a new pattern of programmed cell death that differs from several other forms of regulated cell death in various aspects, including morphology, biochemistry, and immune status (Table 1).

**Table 1:** Comparison of different forms of programmed cell death.

**Cell death**	**Morphological features**	**Biochemical changes**	**Immune status**
Ferroptosis	Mitochondria shrinkage, increased mitochondria membrane density, crista destruction, and outer membrane rupture, but not nucleus morphological changes	Lipid peroxidation and iron overload	Pro-inflammatory
Apoptosis	Cell shrinkage, chromatin condensation, plasma membrane blebbing without rupture, formation of apoptotic bodies, cytoskeletal disintegration	DNA fragmentation	Anti-inflammatory (mostly)
Necroptosis	Cytoplasm and organelles swelling, formation of necrosome, plasma membrane rupture, and release of cell contents	ROS production, random degradation of DNA, damage-associated molecular patterns (DAMPs) release, R1PK1, R1PK3 and MLKL phosphorylation	Anti-inflammatory
Autophagy	Formation of double- membraned autophagic vesicles, normal membrane and nucleus	Increased lysosomal activity, LC3-I to LC3-II conversion, P62 degradation	Anti-inflammatory (mostly)
Pyroptosis	Cytoplasm swelling, formation of pyroptotic bodies, plasma membrane rupture, release of cell contents, and unaffected mitochondrial integrity	Activation of caspase and GSDMD, pro-inflammatory factors release	Pro-inflammatory (mostly)

**Figure 1:**
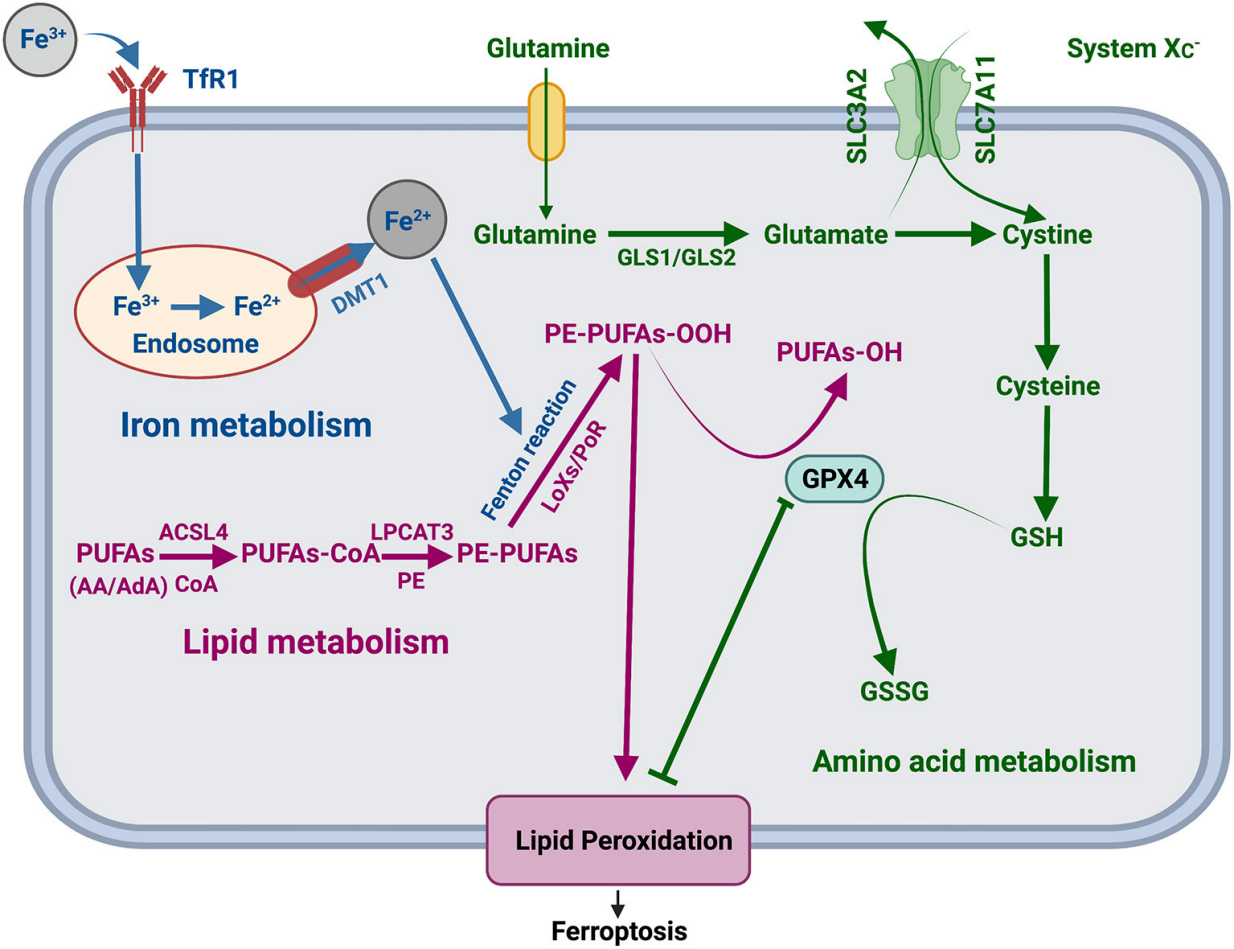
Regulatory mechanism of ferroptosis. TfR1, transferrin receptor 1; DMT1, divalent metal transporter 1; LOXs, lipoxygenases; POR, cytochrome P450 oxidoreductase; PUFAs, polyunsaturated fatty acids; AA, arachidonic acid; AdA, adrenal acid; CoA, coenzyme A; PE, phosphatidylethanolamine; ACSL4, acyl-CoA synthetase long-chain family member 4; LPCAT3, lysophosphatidylcholine acyltransferase 3; SLC7A11, solute carrier family 7 member 11; SLC3A2, solute carrier family 3 member 2; GSH, glutathione; GSSG, glutathione disulfide.

Recently, several studies have found various significant factors of ferroptosis and revealed a range of complex regulatory mechanisms in the progression of ferroptosis involving iron metabolism, lipid metabolism, and amino acid metabolism (Figure 1). In the iron metabolism pathways, transferrin receptor 1 (TfR1) transport extracellular Fe^3+^ to the nucleus and convert it into Fe ^2+^, which is released from the nucleus through divalent metal transporter 1 (DMT1), triggering the Fenton reaction, activating lipoxygenases, and promoting the generation of lipid peroxides, resulting in ferroptosis (4, 5). Amino acid metabolism involves vital regulatory factors, including system ${\text{X}}_{\text{C}}^{-}$ (consisting of two subunits SLC3A2 and SLC7A11) (6, 7) and glutathione peroxidase 4 (GPX4). Inhibitors of system ${\text{X}}_{\text{C}}^{-}$ decrease the uptake of cystine and reduce cysteine and suppress glutathione (GSH) production, further inactivating GPX4 (8) and reducing the conversion of GSH to glutathione disulfide (GSSG) (9), which will result in lipid peroxidation and ferroptosis in amino acid metabolism. By activating acyl-CoA synthetase long-chain family member four (ACSL4) and lysophosphatidylcholine acyltransferase three (LPCAT3), polyunsaturated fatty acids (PuFAs) induce lipid peroxidation and promote ferroptosis (10).

Over the years, researches on the link between ferroptosis and clinical diseases have been gradually improved, with cancer and neurodegenerative diseases being the focus (11–19). Recent studies have demonstrated ferroptosis participates in the genesis and development of cardiovascular diseases. We discuss the roles and potential mechanisms of ferroptosis in cardiovascular diseases in this article and hopefully provide an effective strategy for the treatment of cardiovascular diseases.

## Ferroptosis and Cardiovascular Diseases

### Ferroptosis and Cardiomyopathy

Cardiomyopathy is a group of myocardial diseases caused by heterogeneous factors, leading to myocardial and/or cardiac electrical dysfunction, with high mortality (20).

Doxorubicin (DOX), also known as adriamycin, is the second-generation anthracycline chemotherapy drug, a commonly used antitumor agent with fatal cardiotoxicity. Its most serious side effect is cardiomyopathy, called doxorubicin-induced cardiomyopathy (DIC) (21). Tadokoro et al. (22) found that mitochondria-dependent ferroptosis plays an essential role in DIC. DOX down-regulated GPX4 and caused excessive lipid peroxides production in mitochondria through the DOX-Fe^2+^ complex, resulting in mitochondria-dependent ferroptosis. GPX4 overexpression in mitochondria or iron chelates targeting Fe^2+^ can ameliorate doxorubicin-induced ferroptosis. Furthermore, this study showed that apoptosis is also a major form of doxorubicin-induced cardiomyocyte death. And two death forms are independent of each other. The combination of ferrostatin-1 (Fer-1) and zVAD-FMK to inhibit ferroptosis and apoptosis could completely prevent doxorubicin-induced cardiomyocyte death in rats. In addition, Fang et al. (23) showed that DOX significantly up-regulated heme oxygenase-1 (Hmox1) through NF-E2-related factor 2 (NRF2), induced local heme degradation, leading to the release of free iron, and further inducing ferroptosis in mouse myocardial tissue. Zinc protoporphyrin IX (ZnPP), a competitive inhibitor of Hmox1, reduced DOX-induced ferroptosis. These results suggest that Hmox1 plays an important role in doxorubicin-induced ferroptosis and cardiomyopathy. This study also found that ferroptosis inhibitor Fer-1 or dexrazoxane (DXZ) prevented lipid peroxidation and DIC by maintaining mitochondrial function. However, MitoTEMPO, a mitochondria-targeted antioxidant, can alleviate DIC by specifically clearing lipid peroxidation in mitochondria. These studies show that DOX-induced cardiotoxicity is closed with mitochondrial iron overload and subsequent ferroptosis. In 2021, He et al. (24) proved *in vitro* and *in vivo* that ferroptosis, autophagy, and apoptosis are related to DOX-induced cardiotoxicity. Epigallocatechin-3-gallate(EGCG)is a polyphenol compound in green tea and is also a natural antioxidant. EGCG up-regulated AMP-activated protein kinaseα2 (AMPKα2), activated adaptive autophagy, reduced iron deposition, inhibited reactive oxygen species (ROS) overproduction and rectified abnormal lipid metabolism, thereby reversing ferroptosis in DIC. Similarly, in a recent article, Sun et al. (25) demonstrated potent antioxidant melatonin inhibited mitochondrial lipid peroxidation and ameliorated doxorubicin-induced cardiac ferroptosis. In summary, we know that many forms of cell death are involved in DIC, among which ferroptosis is a pivotal one. Thus, targeting ferroptosis might be an effective treatment for DIC in cancer patients.

Diabetic cardiomyopathy (DCM) is defined as a disorder of cardiac structure and function in patients with diabetes in the absence of coronary artery disease, hypertension, valvular heart diseases, and other conventional cardiovascular risk factors (26). Excessive overproduction of ROS is regarded as an essential mechanism for the occurrence and development of diabetic cardiomyopathy (27), and the accumulation of lipid ROS induced ferroptosis (28). Therefore, ferroptosis is more likely to be involved in DCM. Some studies have supported that administration of ferroptotic inhibitors coenzyme Q_10_ and Vitamin E in diabetic animals might protect the myocardium by suppressing oxidative stress (29, 30). GPX4 is one of the crucial regulators of ferroptosis, and GPX4 deficiency induced lipid peroxidation and resulted in myocardial metabolic disturbance in high-fat, high-sucrose diet mice (31). Conversely, GPX4 overexpression could alleviate mitochondrial dysfunction and protect the hearts from diabetic damage (32). A recent study has identified that ferroptosis exerts a pivotal effect on the pathogenesis of DCM. NRF2 agonist sulforaphane inhibited lipid peroxidation via AMPK/NRF2 pathways, which suppressed ferroptosis and prevented DCM (33). These findings suggest that ferroptosis has a substantial impact on DCM.

Sepsis cardiomyopathy is a severe life-threatening complication caused by sepsis (34). Li et al. (35) found ferroptosis is involved in the progression of sepsis cardiomyopathy. Their experiments showed that ferroptotic inhibitor Fer-1 or iron chelates DXZ mitigated lipopolysaccharide (LPS)-induced ferroptotic cell death in sepsis cardiomyopathy model, while ferroptosis inducers sorafenib and erastin exacerbated LPS-induced myocardial injury.

In conclusion, ferroptosis plays a crucial role in the pathogenesis of cardiomyopathy, and ferroptosis inhibitors are expected to be a novel therapeutic strategy for cardiomyopathy.

### Ferroptosis and Atherosclerosis

Atherosclerosis is a chronic inflammatory disease involving the main and middle arteries (36). Martinet et al. (37) suggested that intraplaque hemorrhage, iron deposition, and lipid peroxidation are common pathological features of an advanced stage of human atherosclerotic plaque. Guo et al. (38) have found that overexpression of GPX4 inhibited lipid peroxidation and delayed the pathological process of atherosclerosis in ApoE^−/−^mouse. And lipid peroxidation accumulation is one of the characteristics of ferroptosis, so we speculate that ferroptosis plays an essential role in the initiation and development of atherosclerosis. CD98 heavy chain (CD98hc), also named solute carrier family 3 member 2 (SLC3A2), is a component of the antiporter system ${\text{X}}_{\text{C}}^{-}$. Inhibitor of system ${\text{X}}_{\text{C}}^{-}$ triggered endoplasmic reticulum stress and resulted in ferroptosis, while the expression of CD98hc in vascular smooth muscle cells contributed to the stable formation of atherosclerotic plaque (39, 40). There is direct evidence that ferroptosis occurs in the development of atherosclerosis. Ferroptotic inhibitor Fer-1 delayed the progression of atherosclerosis by reducing endothelial dysfunction, lipid peroxidation and iron content in mouse aortic endothelial cells (41). It is well known that diabetes can be complicated with vascular diseases, which include atherosclerosis. A study by Meng et al. (42) indicated that ferroptosis is involved in the occurrence and development of atherosclerosis in diabetes mellitus. In the cell models treated with high glucose, and high lipids, Hmox1 deficiency reduced iron overload, ROS production and lipid peroxidation to inhibit ferroptosis in endothelial cells. Hmox1 may be a therapeutic target for diabetic atherosclerosis. Based on those studies, we know that ferroptosis has an essential effect on atherosclerosis. Targeting ferroptosis may provide new ideas for the treatment of atherosclerosis.

### Ferroptosis and Acute Myocardial Infarction

The clinical definition of AMI refers to myocardial injury with abnormal cardiac biomarkers detected in the condition of acute myocardial ischemia (43). Park et al. (44) found that the down-regulation of GPX4 induced ferroptosis during AMI, resulting in cardiomyocyte death and myocardial injury. Baba et al. (45) showed that mechanistic target of rapamycin (mTOR) suppressed cell death, ferroptosis and improved left ventricular remodeling by reducing the production of ROS. MiR-23a-3p is a kind of enriched miRNAs in exosomes derived from mesenchymal stem cells (MSCs) (46). It was reported that DMT1 is a miR-23a-3p target gene. Ferroptosis occurred in the hypoxic cardiomyocytes and infarcted myocardium. MSCs exosomes derived from human umbilical cord blood inhibited ferroptosis via miR-23a-3p/DMT1 axis and mediated myocardial repair in AMI mice (47). In the above studies, ferroptosis has been implicated in the initiation and development of AMI. Inhibition of ferroptosis has been provide novel tactics for the precise treatment of myocardial infarction. Meanwhile, Through machine learning, Huang et al. (48) filtered out ferroptosis-related genes (FRGs) specifically expressed in the peripheral blood of AMI patients. In this study, they also proposed a diagnostic model composed of mitogen-activated protein kinase 3 (MAPK3), WD repeat domain phosphoinositide-interacting protein 2 (WIPI2) and voltage-dependent anion channel three (VDAC3) and provided a new direction for early diagnosis of AMI.

Since diabetes mellitus significantly inhibits the establishment of collateral circulation of ischemic myocardium, aggravating myocardial injury, patients with diabetes comorbiditied with AMI have higher incidence and mortality of coronary heart disease (49). Diabetes increases ROS production in the infarcted myocardium (50), and ROS are considered as essential signals of ferroptosis (51). We hypothesize that ferroptosis might be involved in the pathological process of diabetes comorbiditied with AMI. However, it has not been reported explicitly whether ferroptosis participates in diabetes comorbiditied with AMI, and further studies are needed.

### Ferroptosis and Myocardial Ischemia/Reperfusion Injury

Myocardial ischemia/reperfusion injury (I/RI) refers to the pathological process of aggravated myocardial damage caused by reperfusion within a certain period of time after partial or complete acute occlusion of coronary artery. Tang et al. (52) proposed that up-regulation of ubiquitin-specific protease 7 (USP7) activated the protein 53 (p53)/TfR1 pathway to promote ferroptosis in the I/RI rat model. Increased oxidized phosphatidylcholines (OxPCs) caused mitochondrial dysfunction and disrupted calcium transients and resulted in extensive cardiomyocyte death via ferroptosis during myocardial I/RI. Intervention to OxPCs could prevent ferroptosis in I/RI patients (53). These findings supported that ferroptosis might play a significant role in the pathogenesis of myocardial I/RI. Pretreating mice with ferroptotic inhibitor Fer-1, DXZ or liproxstatin-1 (Lip-1) could alleviate myocardial injury after ischemia/reperfusion (23, 54). The latter was mainly achieved by reducing mitochondrial ROS production, increasing GPX4 level, and decreasing voltage-dependent anion channel 1 (VDAC1) level (54). Anthocyanins can be found in most plants and cyanidin-3-glucoside (C3G) is a major type of anthocyanins. Anthocyanins have strong antioxidant activity, which can effectively scavenge free-radical and protect the heart (55). C3G suppressed the promotion of ras synthetic lethal 3 (RSL3) on ferroptosis. C3G reduced the Fe^2+^content, down-regulated TfR1 and up-regulated ferritin heavy chain1 (FTH1), inhibited ferroptosis and alleviated myocarial injury in I/RI models (56). Likewise, Xanthohumol (XN) isolated from Humulus lupulus had also been shown to protect ischemic/reperfusion myocardium from ferroptosis (57). Besides, exosomal long noncoding RNA (lncRNA) MIR9-3 host gene (Mir9-3hg) derived from bone MSCs mitigated ferroptosis in I/RI mice by regulating pumilio RNA binding family member two (Pum2)/peroxiredoxin 6 (PRDX6) axis and showed cardioprotective effects both *in vitro* and *in vivo* (58). These exciting findings have further broadened therapeutic approaches for ferroptosis in I/RI.

Recent studies have demonstrated the pathological process of diabetic I/RI is relevant to ferroptosis. Wang et al. (59) discovered that diabetes exacerbated I/RI via decreasing AMPK, inducing oxidative stress associated with NADPH oxidase 2 (NOX2) and programmed cell death including ferroptosis. Meanwhile, Li et al. (60) found that restraining ferroptosis could reduce endoplasmic reticulum stress and oxidative stress damage and delay the progression of diabetic I/RI. Nevertheless, the role of ferroptosis in diabetes I/RI needs to be better elucidated.

Ferroptosis also participates in I/RI related to heart transplantation. Ferroptosis mediated I/RI after heart transplantation by recruiting neutrophils to the transplanted heart. Inhibition of ferroptosis before transplantation can alleviate reperfusion injury, reduce left ventricular remodeling, and improve the prognosis of heart transplant recipients (61).

### Ferroptosis and Heart Failure

Heart failure is a set of clinical syndromes in which cardiac output is inadequate due to various structural and functional abnormalities of the heart (62). The loss of cardiomyocytes plays a crucial part in the development of heart failure. Programmed cell death, such as autophagy and ferroptosis, occurs in the heart failure stage. Knockdown of toll-like receptor 4 (TLR4) or NADPH oxidase 4 (NOX4) restrained ferroptosis and autophagy, which attenuated the loss of cardiomyocytes and delayed the progression of heart failure (63). Moreover, ferroptosis has been observed in heart failure resulted from pressure overload. The model of heart failure was established by aortic coarctation in this research. Antioxidant puerarin could inhibit ferroptosis via increasing GPX4 and ferritin heavy chain 1 (FTH1), and down-regulating expression of NOX4, which could improve cell viability in rats, reduce death of H9C2 cardiomyocytes treated with erastin or isoproterenol (ISO) and retard the development of heart failure (64). Nitenberg et al. (65) demonstrated abnormal myocardial iron probably exists in diabetic heart failure. Iron chelator deferoxamine can improve coronary microcirculation in patients with type two diabetes by suppressing the increase of oxygen radicals, which may be a novel target for reversing deterioration of cardiac function in patients with diabetic heart failure. Nevertheless, the toxicity and short half-life of deferoxamine affect its application in improving cardiac function for clinical patients with diabetic heart failure. Thus, the role of ferroptosis in heart failure remains to be further studied.

### Ferroptosis and Other Cardiovascular Diseases

Hypertension is a common cardiovascular disease. Currently, there are few works on the relationship between hypertension and ferroptosis. A research by Yang et al. (66) showed that reductions of GPX4 and GSH in the brains of hypertensive rats led to lipid peroxidation and iron overload, inducing hypertensive brain injury. Elabela is an endogenous ligand for apelin receptor, which is primarily expressed in the cardiac microvascular endothelial cells (CMVECs). Zhang et al. (67) studied the effect of elabela on hypertension. They found that elabela inhibited cardiac oxidative stress, inflammation, fibrosis, and ferroptosis in Angiotensin II (Ang-II) treated CMVECs and hypertensive mice to suppress hypertensive ventricular remodeling. Hence, we guess that ferroptosis might be involved in hypertension and result in the damage to hypertensive target organs.

Aortic dissection (AD), also known as aortic dissecting aneurysm (ADA), is a type of cardiovascular diseases with high mortality (68). Zou et al. (69) revealed that ferroptosis is an important pathological mechanism of Stanford type A aortic dissection (TAAD). Some ferroptosis-related genes mediated ferrptosis in cells and influenced the development of TAAD. Smooth muscle cell (SMC) loss is an important mechanism of aortic dissection. Ferroptosis participated in SMC loss and AD progression. BRD4770 is a new ferroptosis inhibitor, which suppressed inflammatory response, reduced lipid peroxidation and inhibited ferroptosis in SMC of AD mice to prevent the formation of aortic dissection (70, 71).

In addition, recent studies have indicated a possible link between ferroptotic death and arrhythmia. Iron overload caused the occurrence of arrhythmia via promoting mitochondrial ROS generation and membrane potential depolarization, and mitochondrial dysfunction is one of the main characteristics of ferroptosis (72). Frequent alcohol consumption is known to increase the risk of atrial fibrillation (73). Regular drinking promoted ferroptosis via iron overload and increased the incidence of atrial fibrillation. Ferroptosis inhibitor Fer-one, reduced the susceptibility to atrial fibrillation induced by frequent drinking in mice (74). Hence, we supposed that ferroptotic cell death might be a latent target for arrhythmia therapy in the future.

## Discussion

Ferroptosis is a novel regulated cell death, which has received much attention in recent years. We discuss the roles of ferroptosis in cardiomyopathy, atherosclerosis, acute myocardial infarction, ischemia, and reperfusion injury, heart failure, hypertension, arrhythmia and aortic dissection in this review (Table 2). But the roles of ferroptosis in other cardiovascular diseases, including valvular heart disease, have been rarely studied, which require further researches. Besides, except for iron chelators DXZ and deferiprone (DFP) authorized by FDA are used in treating DIC and AMI (75, 76), a majority of researches of ferroptosis in cardiovascular diseases have only been confirmed in the cell and animal models, with relatively limited clinical evidence. Thus, clinical investigations are essential for the application of ferroptosis in cardiovascular diseases. Furthermore, ferroptotic inhibitors are greatly limited in the human body due to their toxicity, instability and short half-life. And it is urgent to develop non-toxic and long-acting inhibitors targeting ferroptosis.

**Table 2:** The role of ferroptosis in various cardiovascular diseases.

**Diseases**	**Characteristics or changes**	**Pathways or signals**	**References**
DIC	Excess lipid peroxides production in mitochondria	Down-regulation of GPX4 expression	Tadokoro et al. (22)
DIC	Up-regulation of Hmox1 expression	NRF2/Hmox1 pathway	Fang et al. (23)
DCM	Lipid peroxidation	Advanced Glycation end-products (AGEs) inhibited SLC7A11 expression and ferritin, decreased GSH expression and increased unstable iron levels.	Wang et al. (33)
Sepsis cardiomyopathy	Iron overload and excessive ROS in mitochondria	NCOA4 expression increased, interacted with ferritin, activated SFXN1 expression, and transferred Fe^2+^ to mitochondria	Li et al. (35)
Diabetic Atherosclerosis	Iron overload, ROS increased, down-regulation of GPX4 and SCL7A11, lipid peroxidation and together resulted in ferroptosis in endothelial cells	Hmox1 increased	Meng et al. (42)
AMI	Accumulation of lipid peroxides	Down-regulation of GPX4	Park et al. (44)
AMI	GSH level decreased, iron deposition, Fe^2+^ level increased, excessive lipid peroxides and ROS	DMT1 overexpression	Song et al. (47)
I/RI	Up-regulation of USP7, p53 and TfR1	USP7 / p53 / TfR1 pathway	Tang et al. (52)
I/RI	Mitochondrial dysfunction, calcium transients blocked and contractile dysfunction	Loss of GPX4 activity	Stamenkovic et al. (53)
Diabetic I/RI	A increase in myocardial oxidative stress, apoptosis, pyroptosis and ferroptosis	Nox2 activation mediated through AMPK suppression	Wang et al. (59)
Diabetic I/RI	The interaction between endoplasmic reticulum stress and ROS caused cardiomyocytes injury	ATF4-CHOP pathway	Li et al. (60)
I/RI related to heart transplantation	Neutrophils recruitment to impaired myocardium	TLR4/TRIF pathway	Li et al. (61)

*DIC, doxorubicin-induced cardiomyopathy; GPX4, glutathione peroxidase 4; Hmox1, heme oxygenase-1; NRF2, NF-E2-related factor 2; DCM, Diabetic cardiomyopathy; AGEs, advanced glycation end-products; ROS, reactive oxygen species; NCOA4, nuclear receptor coactivator 4; SFXN1, siderofexin; SCL7A11, solute carrier family 7 member 11; AMI, acute myocardial infarction; GSH, glutathione; DMT1, divalent metal transporter 1; I/RI, ischemia/reperfusion injury; USP7, ubiquitin-specific protease 7; p53, protein 53; TfR1, transferrin receptor 1; Nox2, NADPH oxidase 2; AMPK, AMP-activated protein kinase; ATF4, Activating transcription factor 4;CHOP, C/EBP homologous protein; TLR4, toll-like receptor 4; TRIF, TIR domain-containing adapter-inducing interferon-β*.

A series of researches showed that ferroptosis and other types of programmed cell death take part in cardiovascular diseases together (22, 24, 59, 63). Whether there is a crosstalk between ferroptosis and other cell death forms in various cardiovascular diseases is unclear and needs further researches, which is crucial for reducing cardiomyocyte death and broadening the treatment models of cardiovascular diseases. Liu et al. (77) found that self-assembly indocyanine green-Lecithin (ICG/LECI) can be used to enhance magnetic resonance/ photoacoustic (MR/PA) imaging and reduce iron toxicity, opening the way for personalized diagnosis and treatment for iron overload patients. FRGs specifically expressed in the peripheral blood of AMI patients also provided a new direction for early diagnosis of AMI. However, more attention needs to be paid to the development of testing methods suitable for routine clinical diagnosis of ferroptosis, and the introduction of biomarkers of ferroptosis characteristics is expected to provide helps for the early identification and diagnosis of cardiovascular disease.

In conclusion, ferroptosis plays a key role in the progression of cardiovascular diseases, and the roles of ferroptosis in cardiovascular diseases remain to be further studied. We can anticipate that diagnostic tools and therapeutic drugs based on ferroptosis will greatly help in the diagnosis and treatment of cardiovascular diseases in the future.

## Author Contributions

MS: conceived and designed the review. YuG, WZ, XZ, SZ, JWa, YiG, YL, HL, and JL: collected the literatures. YuG, WZ, and MS: wrote the manuscript. MS, JWu, and YC: reviewed and edited the manuscript. JWu: revised the manuscript and the language. All authors contributed to the article and approved the submitted version.

## Funding

This study was supported by Hainan Science and Technology Project (ZDYF2020123, ZDYF2020027, and ZDKJ2019012), Hainan Province Clinical Medical Center, National Key R & D Plan (2020YFC2004706), and National Natural Science Foundation of China (Fund No. 81500202).

## Conflict of Interest

The authors declare that the research was conducted in the absence of any commercial or financial relationships that could be construed as a potential conflict of interest.

## Publisher's Note

All claims expressed in this article are solely those of the authors and do not necessarily represent those of their affiliated organizations, or those of the publisher, the editors and the reviewers. Any product that may be evaluated in this article, or claim that may be made by its manufacturer, is not guaranteed or endorsed by the publisher.
